# Early Inflammatory Cytokine Expression in Cerebrospinal Fluid of Patients with Spontaneous Intraventricular Hemorrhage

**DOI:** 10.3390/biom11081123

**Published:** 2021-07-30

**Authors:** Wendy C. Ziai, Adrian R. Parry-Jones, Carol B. Thompson, Lauren H. Sansing, Michael T. Mullen, Santosh B. Murthy, Andrew Mould, Saman Nekoovaght-Tak, Daniel F. Hanley

**Affiliations:** 1 Department of Neurology, Johns Hopkins University, Baltimore, MD 21287, USA; 2 Manchester Academic Health Sciences Centre, School of Medical Sciences, The University of Manchester, Salford Royal NHS Foundation Trust, Salford M6 8HD, UK; adrian.parry-jones@manchester.ac.uk; 3 Department of Biostatistics, Johns Hopkins Bloomberg School of Public Health, Baltimore, MD 21287, USA; cthomp45@jhu.edu; 4 Department of Neurology, Yale University, New Haven, CT 06520, USA; lauren.sansing@yale.edu; 5 Department of Neurology, University of Pennsylvania, Philadelphia, PA 19104, USA; Michael.Mullen@uphs.upenn.edu; 6 Department of Neurology, Weill Cornell Medicine, New York, NY 10065, USA; sam9200@med.cornell.edu; 7 Department of Neurology, Division of Brain Injury Outcomes, Johns Hopkins University School of Medicine, Baltimore, MD 21287, USA; amould@jhmi.edu (A.M.); snekoov1@gmail.com (S.N.-T.); dhanley@jhmi.edu (D.F.H.)

**Keywords:** intracerebral hemorrhage, intraventricular hemorrhage, neuroinflammation, cytokines, cerebrospinal fluid

## Abstract

We investigated cerebrospinal fluid (CSF) expression of inflammatory cytokines and their relationship with spontaneous intracerebral and intraventricular hemorrhage (ICH, IVH) and perihematomal edema (PHE) volumes in patients with acute IVH. Twenty-eight adults with IVH requiring external ventricular drainage for obstructive hydrocephalus had cerebrospinal fluid (CSF) collected for up to 10 days and had levels of interleukin-1α (IL-1α), IL-1β, IL-6, IL-8, IL-10, tumor necrosis factor-α (TNFα), and C-C motif chemokine ligand CCL2 measured using enzyme-linked immunosorbent assay. Median [IQR] ICH and IVH volumes at baseline (T0) were 19.8 [5.8–48.8] and 14.3 [5.3–38] mL respectively. Mean levels of IL-1β, IL-6, IL-10, TNF-α, and CCL2 peaked early compared to day 9–10 (*p* < 0.05) and decreased across subsequent time periods. Levels of IL-1β, IL-6, IL-8, IL-10, and CCL2 had positive correlations with IVH volume at days 3–8 whereas positive correlations with ICH volume occurred earlier at day 1–2. Significant correlations were found with PHE volume for IL-6, IL-10 and CCL2 at day 1–2 and with relative PHE at days 7–8 or 9–10 for IL-1β, IL-6, IL-8, and IL-10. Time trends of CSF cytokines support experimental data suggesting association of cerebral inflammatory responses with ICH/IVH severity. Pro-inflammatory markers are potential targets for injury reduction.

## 1. Introduction

Intraventricular hemorrhage and ensuing hydrocephalus complicate spontaneous intracerebral hemorrhage in up to 45% of ICH patients, and independently contribute to poor outcomes [1,2,3]. Acute hydrocephalus, mass effect and intracranial hypertension cause the primary damage, whereas the inflammatory response likely contributes to the progression of secondary injury [4].

An aseptic inflammatory response in CSF has been demonstrated in experimental and clinical ICH/IVH [4,5,6], and transient increases in inflammatory cytokines in CSF have been described in small cohorts with aneurysmal SAH [7,8,9,10]. Elevations in serum inflammatory markers following spontaneous ICH have been reported in clinical studies and were found to correlate with both radiographic parameters of brain injury and clinical outcomes [11,12,13,14]. The investigation of inflammatory cytokines from within the central nervous system in ICH is limited predominantly to experimental models, with little information regarding the expression of cytokines within the ventricular cavity [15,16,17]. Cytokines and chemokines have been implicated in inflammatory models of ICH as mediators of increased vascular permeability, inducing edema formation [13,17,18], recruitment of leukocytes to the brain, and in the case of TNF-α to direct cytotoxic effects and activation of other bioactive compounds [19,20]. The cytokine response is a potential therapeutic target in ICH and modifiable with commercially available antibodies. Therefore, understanding the molecular mechanisms underlying secondary injury after clinical ICH could guide the development and application of novel therapies.

In this study, we evaluated the early expression of cytokine levels in CSF among patients with spontaneous IVH with or without parenchymal ICH. We hypothesized that the extravasation of red blood cells into the intraventricular compartment and their subsequent lysis leads to activation of the immune response, resulting in elevated CSF cytokines, and that this response is proportional to IVH volume, and clinical severity

## 2. Materials and Methods

### 2.1. Study Design

This was a prospective three-center study of 28 adults with severe spontaneous IVH requiring an EVD for obstructive hydrocephalus. We excluded patients with ruptured aneurysm, tumors, and other brain lesions which could have contributed to ICH, as well as any patient with bacterial ventriculitis/meningitis (defined by fever and a positive cerebrospinal fluid culture) during the CSF collection period. EVDs were placed as a part of routine clinical care in patients with symptomatic hydrocephalus.

### 2.2. Measurements

CSF and blood samples were collected for analysis daily starting on day 1 and for up to 10 days after ICH/IVH symptom onset, depending on patient clinical status and site. CSF samples were centrifuged immediately at 2000× *g* for 10 min, and the supernatant was stored at −80 °C. Using the validated Luminex assay (R&D System, Minneapolis, MN, USA), we measured concentrations of IL-1α, IL-1β, IL-6, IL-8, IL-10, TNF-α, and CCL2 (aka MCP-1) in CSF. Serum WBC was also recorded for the first 2 days. All biomarker measurements were performed blinded to clinical and neuroimaging findings.

Non-contrast CT scans performed within 24 h of presentation (T0) and at end of CSF collection +/− 24 h (Te) were used to measure IVH, ICH, and PHE volumes by a trained image reader blinded to clinical outcomes using semi-automated, computerized volumetric analysis on an open source DICOM viewer software program for Mac (Osirix v. 4.1, Pixmeo; Geneva, Switzerland). The software adjusts for changes in slice thickness and thereby corrects for different CT techniques across centers. The distinction between hemorrhage and surrounding edema was made according to specific signal density on CT slices, with hyperdensity representing hematoma and hypodensity in a PHE distribution considered to represent brain edema (Hounsfield unit (HU) range of 5 to 33) [21]. Areas of edema were carefully distinguished from periventricular white matter disease. This technique has been previously validated with high inter- and intra-observer reliability [21]. Absolute PHE volume was determined by subtracting the hemorrhage volume from the total lesion volume. Relative PHE volume was calculated by dividing absolute PHE by hematoma volume to control for ICH size, where ICH volume > 0.

Patient demographics and comorbidities were recorded at time of enrollment. Maximum temperature was recorded on each day of CSF collection. Any clinically proven infection during the first week of admission was collected. Informed consent was provided by the most appropriate surrogate decision maker. This study was approved by the Institutional Review Board of each center.

### 2.3. Statistics

The primary outcome measure of the study was serial levels of inflammatory mediators in the ventricular fluid. We substituted half of the LLOD for cytokine values below the detection limits of each assay (IL-1α: 1.38; IL-1β: 0.79; IL-10: 0.82; TNFα: 1.87; IL-6: <9.0; IL-8: <6.4; CCL2: <5.6 pg/mL). Due to small sample size and lack of daily CSF levels in all subjects, we report levels per 2-day interval where either a single level or the mean of the level for both days was used for analysis (if one level was at LLOD, the higher level was used). Associations between peak mean concentrations of cytokines for days 1–2 through days 9–10 and: ICH, IVH and PHE and relative PHE volume at admission (T0) and at end of collection (Te) if a corresponding CT existed, and with age, and GCS on admission were evaluated. Based on the distributions and availability of daily cytokine values, Spearman correlations were used to describe the associations of the cytokine values and these continuous measures at each day interval. Mann-Whitney tests were used to compare initial/peak cytokine levels with indicators of occurrence of fever (any maximum daily temperature > 38.3 degrees Celsius, elevated peripheral WBC count (>12 × 103), and documented non-CNS infection within first week of admission. Comparisons of cytokine levels between day interval 9–10 with other day intervals were made with a general linear model analysis, taking into account within-patient correlations across day intervals.

As this was an exploratory analysis, no adjustment was made for multiple comparisons. Statistical analyses were performed using STATA (version 16.1, STATA Corp. College Station, TX, USA). All analyses were two-tailed, and statistical significance was determined by *p* < 0.05.

## 3. Results

### 3.1. Availability of Cytokine Measurements

The number of available measurements per patient varied with 5, 5, 8, 8, and 2 patients having 1, 2, 3, 4 and 5 measurements, respectively. Thus, the number of measurements for day intervals 1–2, 3–4, 5–6, 7–8, and 9–10 was 19, 21, 17, 16, and 8, respectively.

### 3.2. Cerebrospinal Fluid Levels of Inflammatory Cytokines

**Figure 1:**
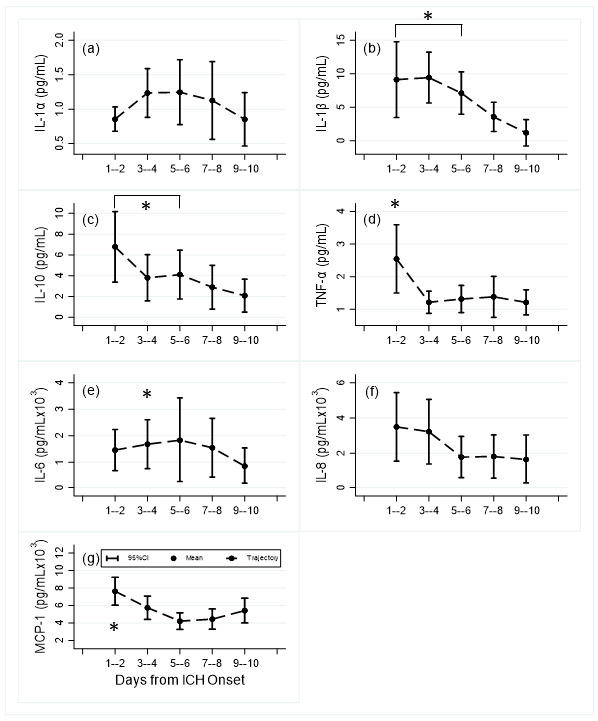
Mean and 95% confidence interval levels of central inflammatory cytokines and chemokines by day time periods: (**a**) IL-1α, (**b**) IL-1β, (**c**) IL-10, (**d**) TNF-α; (**e**) IL-6, (**f**) IL-8, (**g**) MCP-1 (CCL2); (Number of samples): day 1–2 (19); day 3–4 (21); day 5–6 (17); day 7–8 (16); day 9–10 (8). *: *p* < 0.05 compared to day 9–10.

Median [IQR] time from symptom onset to first CSF sample was 1 [1,2,3] days (N = 28). End of sample CT (Te) occurred at 8 [6,7,8,9,10] days (N = 22). Patient characteristics are shown in Table 1. Mean CSF levels of IL-1β, IL-10, TNF-α, IL-6 and CCL2 all showed significant differences in adjusted levels between day 1–2 compared to day 9–10, and for IL-1β and IL-10 between all time points up to day 6 compared to days 9–10 (Figure 1, Table S1). Significant decreases occur after day 1–2 for IL-10, TNF-α, and CCL2, and after day 3–4 for IL-1β and IL-6. For IL1-alpha, and IL-8 levels, although there are patterns of increasing and decreasing cytokine levels across days from admission, the differences in mean levels were not statistically significant.

### 3.3. Cytokine Expression and Correlations with Injury Severity

Table 2 shows Spearman correlation results between the radiographic volumes and cytokine levels. Significant correlations occurred between IVH volume on admission (T0) and levels of IL-6, IL-8, IL-10, and CCL2 and were largest on days 5–6 and 7–8 (Spearman’s rho all >0.50; *p* < 0.05). ICH volume (T0) was significantly correlated with IL-1β, IL-6, IL-10, and CCL2 at day 1–2 post onset. Statistically significant correlations were larger for total blood volume (ICH plus IVH) than for each separately, for all cytokines predominantly at day 1–2, except for IL-1α (day 3–4). PHE and/or relative PHE volume at T0 were significantly correlated with elevations of most cytokines on day 1–2 for PHE (except IL-1 and TNF-α), and on day 7–8 or day 9–10 for relative PHE (except TNF-α and CCL2).

### 3.4. Cytokine Expression and Clinical Characteristics

Comparisons of cytokine levels and clinical characteristics are shown in Supplemental Table S2. Day 1–2 levels of IL-10, TNF-α and CCL2 were inversely associated with occurrence of WBC elevation during the first week after admission; day 3–4 levels of IL-1β, IL-6, IL-8 and IL-10 were positively associated with febrile response during the first week. WBC elevation in first week was inversely associated with CLL2 at day 7–8. IL-1α was positively associated with in-hospital mortality (day 3–4) and IL-10 for day 5–6. Higher age was positively associated with TNF-α and inversely with IL-1α, and admission GCS was associated with TNF-α (day 1–2) and IL-8 (day 7–8) (Table S3). There were no significant correlations between cytokine levels and ICH, IVH or PHE volumes at Te (data not shown).

## 4. Discussion

The presence of intraventricular blood induces inflammation, which may be an important mechanism contributing to early and delayed brain injury in spontaneous ICH and is a potential therapeutic target [5,21]. This hypothesis-generating study is one of relatively few to investigate serial cytokine levels in CSF over the acute phase of spontaneous ICH with IVH. We observed dynamic changes in CSF concentrations of several cytokines; baseline ICH volume was associated with cytokine overexpression at day 1–2, baseline IVH volume with cytokine overexpression at day 5–8, and baseline PHE and relative PHE with cytokine overexpression at days 1–2 and 7–8 respectively. Cytokine levels were not associated with these neuroimaging markers at end of CSF collection (Te, 6–10 days after presentation) which may indicate that later expression does not follow secondary injury as well as in the acute phase. Early cytokine elevation was positively associated with fever and inversely associated with peripheral WBC counts within the first week. IL-1α and IL-10, which had significant correlations with total blood volume and relative PHE, were also associated with in-hospital mortality.

In aggressively managed IVH with EVD, the aseptic inflammatory response is reflected by an early, IVH volume dependent elevation in CSF WBC at days 1–3 followed by a gradual slower rate of WBC decline over the next six days [6]. This response mimics that of the CSF cytokine elevations observed in this study. We reported temporal changes in cytokines/chemokines with reference to day 9–10, although levels may still be altered and do not likely reflect baseline CSF levels at this timepoint. Our observations are, however, also consistent with a transient increase in similar CSF inflammatory cytokines (TNF-α, interferon-γ, IL-1α, IL-1β, IL-2, IL-4, and IL-6) over 72 h reported in a small trial of intraventricular tPA for IVH associated with aneurysmal SAH [10]. Levels of IL-1α, TNF-α and IL-6 were relatively higher in CSF from control SAH patients compared to our observed very low levels in spontaneous IVH, whereas IL-1β was higher in our cohort. CSF IL-1β levels over first 7 days were even higher in a report of 41 adults with primary ICH associated with IVH who did not receive tPA [22]. Whether such comparisons are meaningful in a small sample, however, is not clear and may simply reflect sampling variation. Levels of IL-6 were several magnitudes higher in both our and the SAH cohort compared to other cytokines suggesting that the order of magnitude of elevation in CSF cytokines is comparable between studies despite different etiologies of CSF inflammation.

Our study of acute changes in cytokines lacked a control group. However, studies in other disease states which included controls have investigated CSF inflammatory markers and found that most cytokines were present in CSF at concentrations close to or below the lower limit of detection of the assay [23]. In patients with possible normal pressure hydrocephalus, median ventricular CSF concentrations were reported for IL-8 (19.1 [11.0] pg/mL), TNF-alpha (0), and CCL2 (758 [264.0] pg/mL). Other studies have reported lumbar CSF concentrations from controls and report similar levels with CCL2 concentrations of 300–500 pg/mL [24,25]. The cytokine/chemokine concentrations reported in our patients were one to two orders of magnitude larger than these control values indicating significant elevations.

### 4.1. Cytokine Levels and ICH and IVH Volume

In this study, admission ICH volume was larger than IVH volume and was correlated with early CSF cytokine response (day 1–2), whereas admission IVH volume was correlated with CSF cytokine overexpression at later time intervals (days 5–8). As hemorrhage was present in both compartments from diagnostic CT, it is possible that the parenchymal hematoma is an earlier source of CSF inflammation compared to ventricular blood. Fibrinolysis causing gradual release of hematoma breakdown products may also induce inflammation and may explain the delayed association between cytokine elevation and IVH volume. Changes in the levels of cytokines in the CSF may be attributable to the CNS resident cells or to infiltrating immunocytes from the peripheral blood, although the source of cytokines in both the hematoma and CSF currently remain unknown. Possible local sources include blood in the CSF or hematoma, the endothelial cells of the impaired blood-brain or blood-CSF barrier, or activated microglia, astrocytes, and other CNS resident cells. Release from immunocytes either in the parenchymal hematoma which spill over into the CSF, or in the ventricular clot itself appear most consistent with the relationship with ICH and IVH volumes.

Few studies have investigated CSF levels of cytokines following ICH. Kraus et al. [5] found that soluble adhesion molecule (AM) concentrations ICAM-1 (sICAM-1) and VCAM-1 (sVCAM-1) in CSF were maximal within the first hours after onset of basal ganglia ICH, then decreased significantly on day 2 and only slightly increased thereafter. ICH volume was significantly correlated with these levels in ventricular CSF but not in serum leading to the conclusion that the ventricular inflammatory reaction appears to be pronounced early after ICH onset and may be restricted to the central nervous system. Similar to our results, early levels correlated more strongly with radiological/clinical data than did follow-up measurements. From our Figure 1, we can conclude that most study variables diminished over time, and were probably higher before EVD placement.

### 4.2. Comparison of Clinical CSF Cytokine Levels with Experimental Literature

From the experimental ICH literature, it is postulated that microglia, which are among the first cells contributing to the innate immune response, become activated and develop classic pro-inflammatory or alternative anti-inflammatory phenotypes, although this polarization may be an over-simplification [26]. Activated microglia do appear to be the primary source of cytokines, chemokines, and other immunomodulatory molecules in the brain [27] and TGF-β has been shown to reduce the pro-inflammatory responses in microglia and improve outcomes in murine ICH models [28]. In both collagenase-induced and autologous blood-induced models of ICH, the pro-inflammatory cytokines IL-1β, IL-6, TNF [29,30] and inducible nitric oxide synthase (iNOS) [31] mRNA levels are generally elevated in the acute phase, starting to rise as early as 3 h after ICH and peaking at 3 days [16]. Alternative activation markers, including IL-10, start to increase on day 1 after ICH. As we lacked specific timing for our CSF samples relative to symptom onset and samples were delayed until after EVD placement, we were not likely to observe very early discriminatory differences in the production of pro-inflammatory cytokines such as TNF-α, and IL-6, compared to anti-inflammatory cytokines like IL-10. The latter, secreted by both macrophages and microglia as a feedback mechanism, suppresses pro-inflammatory cytokine production, and similar to our CSF levels, is increased early in both blood and brain tissue in patients with ICH [32].

### 4.3. Cytokine Levels and PHE Volume

Neutrophil infiltration and activation leads to the release of a variety of cytokines and chemokines including IL-1α, IL-1β, TNF, IL-6, IL-8, and CCL2, some of which such as IL-1β appear to be associated with development of PHE [17,33]. In our study, all cytokine concentrations with exception of TNF had significant correlations with either PHE, and/or relative PHE on diagnostic CT. Early day 1–2 significant correlations with PHE occurred for cytokines which were also correlated with ICH volume on day 1–2, whereas significant correlations with relative PHE, which controls for the influence of ICH volume on PHE volume, occurred later at day 7–8. Wang et al. reported that serum levels of IL-4, IL-6, and IL-8 were positively correlated with severity of cerebral edema while IL-10 which peaked at 3 days after ICH was negatively correlated [13]. Discordance between CNS and peripheral sampling of cytokines is not unusual, however, and levels of IL-10 may respond differently locally and systemically.

### 4.4. Cytokine Levels and Indicators of Systemic Inflammation

This study found a positive correlation between CSF IL-1β, IL-6, IL-8 and IL-10 at day 3–4 and fever, and an inverse correlation between IL-10, TNF-α, and CCL2 at day 1–2 and systemic pleocytosis in the first week. In clinical ICH, peripheral WBC count has been associated with larger ICH volume, early neurological deterioration, and worse discharge disposition [34] leading to the hypothesis that activation of the peripheral immune system may enhance injury after ICH, and which is also consistent with experimental work [35]. Although we did not have data on individual contributions of leukocyte cell types, it is noteworthy that higher CSF CCL2, the dominant chemokine for monocyte recruitment, was correlated with lower WBC count in peripheral blood at day 1–2, and 7–8. This suggests that CNS cytokine responses at least in CSF may lag behind peripheral immune activation, and therefore is not a contradiction to the considerable evidence that inflammatory brain lesions promote peripheral leukocytosis [36].

### 4.5. Cytokine Levels and Outcomes

Clinical studies have reported associations between serum inflammatory markers in the acute phase of ICH, mainly CCL2, CXCL10, IL-6, IL-11, CRP, fibrinogen, and CSF cytokines with hematoma expansion and early neurological decline [13,14]. Inflammation is a potential therapeutic target, although in two adult cohorts of spontaneous intraventricular hemorrhage, no correlation was found between CSF inflammatory measures (WBC, corrected WBC, and cell index) and poor functional outcome (modified Rankin Score (mRS) 4–6) or mortality at 30 or 180 days after adjustment for other clinical severity factors. [4,6]. These results do not exclude more subtle effects of the inflammatory response.

Antibody-targeted modulation of clinical ICH includes cytokines studied in this report, specifically reduction in ICH-induced brain edema with overexpression of IL-1 receptor antagonist [37] and augmenting anti-inflammatory cytokines, such as IL-10, in brain tissues [37] to polarize microglia into a protective phenotype to minimize secondary brain damage and promote brain repair [38]. The particular patterns of cytokine elevations seen in this cohort do support potential therapeutic impact for both IL-1β and IL-10 based on relatively high levels in the acute phase and significant correlations with all severity indicators (ICH, IVH and PHE/relative PHE). Alternatively, elevated cytokines may only be a marker of disease severity and may be reduced with fever control.

### 4.6. Limitations

This was a small descriptive study which is limited by lack of a control group or plasma concentrations of cytokines. Therefore, we could not examine interactions of systemic inflammation with that in the CNS. CSF expression levels are correlated with parameters of injury and outcomes; however, this study cannot clarify whether these inflammatory cytokines are derived from blood components leaked into the ventricles or originating from an active immune reaction within the brain. The recovery of cytokines from ventricular catheters also suffers from variability in catheter location relative to IVH, CSF drainage and composition, and hospital protocol-based differences in sample collection times. Tractional damage and effects of symptomatic hydrocephalus and its treatment (increase and then decrease of ventricular size) could also be important variables in the observed changes. The lack of adequate paired day-interval measurements, distributions of the cytokine levels and small sample size precluded the use of parametric analyses for most of the analyses. General linear model analyses were performed to account for within-patient correlations where appropriate, recognizing their limitations. Other sources of bias are lack of data from all subjects at all time intervals.

Future research questions include associations of CSF inflammatory responses with intracranial pressure, chronic hydrocephalus, permanent shunt requirement, and cognitive outcome assessment. Despite a lack of statistical power, however, this study demonstrated elevated levels of cytokines in CSF that were positively associated with ICH, PHE and IVH volume and with a systemic febrile response.

## 5. Conclusions

In conclusion, although the clinical and laboratory correlations observed do not indicate causal relationships, these data support the hypothesis that a significant inflammatory host response in the CSF of patients with IVH contributes to the pathogenesis of this disease. One therapeutic consequence of such findings is the proposition that early intraventricular administration of anti-inflammatory or anti-cytokine strategies may mitigate inflammatory complications after IVH.

## Figures and Tables

**Table 1:** Baseline Characteristics and Outcomes.

Characteristic	N = 28
*Demographics:*	
Age, Mean (Standard Deviation)	59.4 (12.7)
Sex (Male/Female)	14M, 14F
Ethnicity	13
White	12
African-American	2
Hispanic	1
Asian	
*Comorbidities:*	
Hypertension	25 (89%)
Diabetes Mellitus	8 (29.6%)
Coronary Artery Disease	0
Prior stroke or ICH	4 (14.3%)
Hyperlipidemia	9 (32.1%)
Alcohol Abuse	6 (21.4%)
Cocaine use	3 (10.7%)
Anticoagulation	4 (14.3%)
*Clinical History:*	
GCS on admission, median [IQR]	9 [6, 11]
ICH volume, median [IQR] (mL)	19.8 [5.8, 48.8]
IVH volume, median [IQR] (mL)	14.3 [5.3, 38]
ICH location: (lobar/basal ganglia/cerebellar/primary IVH)	5/18/3/2
*Complications:*	
Fever (1st 7 days)	17 (60.7%)
WBC elevation (1st 7 days)	17 (60.7%)
Non-CNS infection	11 (39.3%)
Dead at discharge	10 (35.7%)

Abbreviations: ICH: intracerebral hemorrhage; GCS: Glasgow Coma Scale; IVH: intraventricular hemorrhage; IQR: interquartile range; WBC: white blood cell count; CNS: central nervous system.

**Table 2:** Associations of Radiographic Volumes with Cerebrospinal Fluid Cytokine Levels in Intraventricular Hemorrhage Patients by Days Post Hemorrhage Onset.

Days Post Hemorrhage Onset											
		1–2 (N = 19, 16) ^a^		3–4 (N = 21, 17)		5–6 (N = 17, 14)		7–8 (N = 16, 13)		9–10 (N = 8, 6)	
Cytokine	Radiographic Variables ^b^	Corr ^c^	P ^d^	Corr	*p*	Corr	*p*	Corr	*p*	Corr	*p*
IL-1alpha	ICH Volume (mL)	0.34	0.15	0.34	0.13	0.20	0.44	0.15	0.58	−0.25	0.55
(pg/mL)	IVH Volume (mL)	−0.25	0.30	0.23	0.32	−0.18	0.50	0.22	0.41	0.08	0.85
	PHE Volume (mL)	**0.44**	**0.06**	0.38	0.09	0.27	0.30	0.30	0.25	−0.25	0.55
	Relative PHE Volume (%)	0.11	0.69	0.08	0.76	−0.23	0.43	**0.62**	**0.02**	0.13	0.80
	Total Volume ^e^ (mL)	0.07	0.77	**0.50**	**0.02**	−0.04	0.88	0.10	0.71	−0.41	0.31
IL-1beta	ICH Volume (mL)	**0.51**	**0.03**	0.09	0.68	0.24	0.35	0.18	0.50	−0.18	0.67
(pg/mL)	IVH Volume (mL)	0.10	0.68	**0.44**	**0.049**	0.09	0.74	0.22	0.42	−0.07	0.87
	PHE Volume (mL)	**0.43**	**0.07**	0.03	0.90	0.24	0.34	0.32	0.22	0.04	0.93
	Relative PHE Volume (%)	−0.35	0.19	−0.20	0.43	−0.16	0.58	**0.60**	**0.03**	0.60	0.21
	Total Volume (mL)	**0.60**	**0.006**	0.34	0.14	0.25	0.34	0.17	0.52	−0.48	0.23
IL-10	ICH Volume (mL)	**0.51**	**0.03**	−0.18	0.43	−0.20	0.45	0.07	0.79	−0.25	0.56
(pg/mL)	IVH Volume (mL)	0.06	0.80	**0.48**	**0.03**	**0.63**	**0.007**	0.39	0.14	−0.17	0.69
	PHE Volume (mL)	**0.48**	**0.04**	−0.29	0.19	−0.11	0.67	0.21	0.43	−0.02	0.97
	Relative PHE Volume (%)	−0.18	0.52	−0.34	0.19	0.33	0.25	0.44	0.13	**0.94**	**0.005**
	Total Volume (mL)	**0.64**	**0.003**	0.41	0.06	0.34	0.18	0.20	0.47	−0.61	0.11
TNF-alpha	ICH Volume (mL)	0.42	0.08	−0.01	0.95	−0.06	0.83	−0.48	0.06	−0.50	0.21
(pg/mL)	IVH Volume (mL)	0.22	0.37	0.26	0.26	0.08	0.77	0.25	0.35	0.25	0.55
	PHE Volume (mL)	0.28	0.25	−0.08	0.73	−0.14	0.58	−0.44	0.09	−0.50	0.21
	Relative PHE Volume (%)	−0.50	0.05	−0.10	0.70	−0.40	0.16	0.13	0.67	NA	NA
	Total Volume (mL)	**0.71**	**0.001**	0.27	0.23	0.03	0.92	−0.24	0.38	−0.25	0.55
IL6	ICH Volume (mL)	**0.57**	**0.01**	0.01	0.95	−0.09	0.74	0.24	0.37	0.14	0.73
(pg/mL × 10^3^)	IVH Volume (mL)	0.00	1.00	**0.49**	**0.03**	**0.54**	**0.03**	0.27	0.31	−0.52	0.18
	PHE Volume (mL)	**0.48**	**0.04**	−0.03	0.88	0.04	0.88	0.38	0.14	0.36	0.38
	Relative PHE Volume (%)	−0.24	0.36	−0.13	0.63	0.35	0.21	**0.64**	**0.02**	**0.94**	**0.005**
	Total Volume (mL)	**0.55**	**0.01**	0.32	0.16	0.30	0.25	0.29	0.28	−0.40	0.32
IL8	ICH Volume (mL)	0.39	0.10	0.04	0.87	−0.16	0.53	−0.16	0.55	−0.25	0.55
(pg/mL × 10^3^)	IVH Volume (mL)	0.14	0.57	**0.49**	**0.03**	**0.56**	**0.02**	**0.65**	**0.007**	−0.02	0.96
	PHE Volume (mL)	0.35	0.14	−0.02	0.94	−0.09	0.73	0.00	1.00	−0.04	0.93
	Relative PHE Volume (%)	−0.12	0.65	−0.12	0.66	0.20	0.48	**0.60**	**0.03**	0.71	0.11
	Total Volume (mL)	**0.52**	**0.02**	0.29	0.20	0.22	0.39	0.14	0.59	−0.43	0.29
CCL2	ICH Volume (mL)	**0.51**	**0.03**	−0.07	0.76	−0.11	0.67	−0.05	0.85	−0.11	0.80
(pg/mL × 10^3^)	IVH Volume (mL)	−0.13	0.61	0.43	0.05	0.27	0.29	**0.73**	**0.001**	0.07	0.87
	PHE Volume (mL)	**0.53**	**0.02**	−0.17	0.46	−0.10	0.69	−0.01	0.97	−0.23	0.59
	Relative PHE Volume (%)	−0.12	0.66	−0.12	0.66	−0.01	0.97	0.22	0.47	−0.09	0.87
	Total Volume (mL)	**0.56**	**0.01**	0.13	0.59	−0.14	0.59	0.32	0.22	0.36	0.39

^a^ (N = x, y), where x is number of patients at this time point and y is number of patients for Relative PHE volume, where ICH Volume > 0. ^b^ Measurements at T0 (Radiographic Volumes). ^c^ Spearman Correlation. ^d^ *p*-values are bold if *p* < 0.05. ^e^ Total Volume = ICH Volume + IVH Volume. Abbreviations: ICH, intracerebral hemorrhage; IVH, intraventricular hemorrhage; PHE, perihematomal edema; GCS, Glasgow Coma Scale. *p*-value: 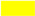 *p* < 0.005 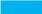 *p* < 0.05 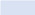 0.05 ≤ *p* ≤ 0.10.

## Data Availability

Not applicable.

## Supplementary Materials

The following are available online at https://www.mdpi.com/article/10.3390/biom11081123/s1, Table S1: Analysis of Comparisons of Cytokine Levels to Day 9–10 Levels in Figure 1, Table S2: Comparisons of Cerebrospinal Fluid Cytokine Levels by Clinical Characteristics for Intraventricular Hemorrhage Patients by Days Post-Hemorrhage Onset, Table S3: Associations of Age and Glasgow Coma Scale with Cerebrospinal Fluid Cytokine Levels in Intraventricular Hemorrhage Patients by Days Post Hemorrhage Onset.
