# Supplementary material for: Early Inflammatory Cytokine Expression in Cerebrospinal Fluid of Patients with Spontaneous Intraventricular Hemorrhage
Source: Biomolecules. 2021 Jul 30;11(8):1123. doi: 10.3390/biom11081123 (PMC8394793; doi:10.3390/biom11081123)
Supplement: Supplementary file 1 [file biomolecules-11-01123-s001.zip › biomolecules-1288596-supplementary.pdf]

Supplemental Tables: Ziai et al., Early inflammatory cytokine expression in cerebrospinal fluid of patients with spontaneous intraventricular hemorrhage

Table S1. Analysis of Comparisons of Cytokine Levels to Day 9-10 Levels in Figure 1

Table S2. Comparisons of Cerebrospinal Fluid Cytokine Levels by Clinical Characteristics for Intraventricular Hemorrhage Patients by Days Post-Hemorrhage Onset

Table S3. Associations of Age and Glasgow Coma Scale with Cerebrospinal Fluid Cytokine Levels in Intraventricular Hemorrhage Patients by Days Post Hemorrhage Onset.

**Table S1. Analysis of Comparisons of Cytokine Levels to Day 9-10 Levels in Figure 1**									
Days 9-10		Cytokine	IL-1alpha (pg/mL)	IL-1beta (pg/mL)	IL-10 (pg/mL)	TNF-alpha (pg/mL)	IL-6 (pg/mLx10^3)	IL-8 (pg/mLx10^3)	CCL2 (pg/mLx10^3)
		Mean (95%CI)[a]	0.86 (0.49, 1.23)	1.18 (-0.80, 3.15)	2.07 (0.48, 3.65)	1.21 (0.82, 1.60)	852 (168, 1536)	1652 (263, 3040)	5423 (4009, 6838)
Days 1-2									
		Mean (95%CI)[a]	0.85 (0.68, 1.03)	9.12 (3.46, 14.78)	6.74 (3.35, 10.14)	2.55 (1.50, 3.59)	1458 (680, 2235)	3502 (1557, 5448)	7640 (6042, 9238)
	Difference[b]	Mean (95%CI)	0.01 (-0.41, 0.43)	**-7.94 (-13.48, -2.41)**	**-4.68 (-8.11, -1.25)**	**-1.34 (-2.37, -0.31)**	-606 (-1799, 588)	-1850 (-3805, 104)	**-2217 (-4330, -103)**
		P	0.96	**0.005**	**0.008**	**0.01**	0.32	0.06	**0.04**
Days 3-4									
		Mean (95%CI)[a]	1.14 (0.87, 1.41)	9.42 (5.63, 13.21)	3.80 (1.58, 6.02)	1.21 (0.87, 1.55)	1679 (755, 2603)	3228 (1396, 5060)	5751 (4419, 7083)
	Difference[b]	Mean (95%CI)	-0.28 (-0.79, 0.23)	**-8.24 (-12.34, -4.15)**	**-1.73 (-3.25, -0.22)**	-0.002 (-0.36, 0.36)	**-827 (-1625, -30)**	-1576 (-3711, 558)	-328 (-2006, 1351)
		P	0.28	**<0.001**	**0.03**	0.99	**0.04**	0.15	0.70
Days 5-6									
		Mean (95%CI)[a]	1.22 (0.75, 1.69)	7.11 (3.95, 10.27)	4.06 (1.72, 6.40)	1.31 (0.90, 1.73)	1831 (231, 3431)	1789 (620, 2959)	4213 (3260, 5167)
	Difference[b]	Mean (95%CI)	-0.36 (-0.98, 0.26)	**-5.94 (-9.78, -2.09)**	**-2.00 (-3.87, -0.12)**	-0.10 (-0.27, 0.07)	-979 (-2306, 348)	-138 (-1084, 809)	1210 (-199, 2619)
		P	0.26	**0.002**	**0.04**	0.24	0.15	0.78	0.09
Days 7-8									

		Mean (95%CI)[a]	1.08 (0.53, 1.63)	3.55 (1.37, 5.73)	2.88 (0.79, 4.98)	1.38 (0.75, 2.01)	1546 (437, 2654)	1821 (595, 3047)	4447 (3288, 5605)
	Difference[b]	Mean (95%CI)	-0.22 (-0.96, 0.52)	-2.38 (-5.63, 0.88)	-0.82 (-2.08, 0.44)	-0.17 (-0.53, 0.19)	-693 (-1669, 282)	-169 (-997, 658)	977 (-510, 2464)
		P	0.57	0.15	0.20	0.36	0.16	0.69	0.20

Footnotes:

[a] Mean (95%CI for a specific day period) is based on a general linear model, accounting for within-patient correlations (See Figure 1)

[b] Mean (95%CI) and P value for difference from Days 9-10 are based on a general linear model, accounting for within-patient correlations

Table S2. Comparisons of Cerebrospinal Fluid Cytokine Levels by Clinical Characteristics for Intraventricular Hemorrhage Patients by Days Post-Hemorrhage Onset

		**Days Post-Hemorraghic Onset**														
		**1-2 (N=19)[a]**			**3-4 (N=21)**			**5-6 (N=17)**			**7-8 (N=16)**			**9-10 (N=8)**		
**Cytokine**	**Clinical Characteristics**	**N**	**Median (IQRb)**	**pc**	**N**	**Median (IQR)**	**p**	**N**	**Median (IQR)**	**p**	**N**	**Median (IQR)**	**p**	**N**	**Median (IQR)**	**p**
**IL-1alpha**	Fever in 1st week - Yes	10	0.7 (0.0)	0.92	14	0.7 (1.0)	0.92	12	0.7 (0.4)	0.94	11	0.7 (0.8)	0.59	7	0.7 (0.0)	1.00
**(pg/mL)**	Fever in 1st week - No	9	0.7 (0.0)		7	0.7 (1.2)		5	0.7 (0.7)		5	0.7 (0.0)		1	0.7 (0.0)	
	WBC elevation in 1st week - Yes	11	0.7 (0.0)	0.75	14	0.7 (1.4)	0.91	11	0.7 (1.3)	0.42	10	0.7 (0.8)	0.43	8		NA
	WBC elevation in 1st week - No	8	0.7 (0.4)		7	0.7 (1.0)		6	0.7 (0.0)		6	0.7 (0.0)		0		
	Non-CNS infection - Yes	7	0.7 (0.0)	0.91	10	0.7 (0.8)	0.69	8	0.7 (0.7)	0.97	8	0.7 (0.4)	0.47	5	0.7 (0.0)	1.00
	Non-CNS infection - No	12	0.7 (0.0)		11	0.7 (1.2)		9	0.7 (0.4)		8	0.7 (0.0)		3	0.7 (0.0)	
	Mortaily at discharge - Yes	8	0.7 (0.9)	0.12	5	**2.1 (0.8)**	**0.02**	3	0.7 (2.2)	0.77	3	0.7 (0.8)	0.98	3	0.7 (0.0)	1.00
	Mortaily at discharge - Yes	11	0.7 (0.0)		16	**0.7 (0.2)**		14	0.7 (0.4)		13	0.7 (0.0)		5	0.7 (0.0)	
**IL-1beta**	Fever in 1st week - Yes	10	4.9 (8.3)	0.72	14	**10.2 (14.2)**	**0.04**	12	6.6 (7.3)	0.82	11	4.0 (8.6)	0.07	7	2.1 (3.4)	0.28
**(pg/mL)**	Fever in 1st week - No	9	3.2 (2.6)		7	**2.3 (3.6)**		5	2.8 (3.5)		5	1.8 (1.0)		1	1.0 (0.0)	

	WBC elevation in 1st week - Yes	11	3.1 (3.7)	0.08	14	4.6 (11.1)	0.47	11	5.6 (6.9)	0.94	10	3.8 (8.8)	0.43	8		NA
	WBC elevation in 1st week - No	8	7.0 (8.3)		7	1.2 (19.2)		6	5.1 (8.7)		6	2.2 (1.9)		0		
	Non-CNS infection - Yes	7	4.7 (7.8)	0.91	10	4.0 (11.3)	0.99	8	4.3 (5.3)	0.21	8	3.9 (2.5)	0.33	5	1.8 (0.4)	0.79
	Non-CNS infection - No	12	3.6 (8.8)		11	4.8 (15.7)		9	9.1 (12.5)		8	1.8 (5.0)		3	4.6 (4.1)	
	Mortaily at discharge - Yes	8	4.6 (6.2)	0.49	5	4.5 (2.2)	1.00	3	9.1 (25.8)	0.76	3	1.6 (11.8)	0.61	3	1.0 (4.7)	0.39
	Mortaily at discharge - Yes	11	3.1 (9.2)		16	4.5 (15.0)		14	4.2 (5.5)		13	3.5 (2.8)		5	2.1 (2.7)	
**IL-10**	Fever in 1st week - Yes	10	2.1 (6.6)	0.39	14	**2.6 (4.7)**	**0.04**	12	3.4 (5.9)	0.19	11	1.5 (5.0)	0.19	7	1.7 (3.3)	0.50
**(pg/mL)**	Fever in 1st week - No	9	5.4 (14.8)		7	**0.4 (0.3)**		5	1.2 (0.5)		5	0.6 (0.6)		1	0.4 (0.0)	
	WBC elevation in 1st week - Yes	11	**1.5 (2.9)**	**0.007**	14	0.5 (3.3)	0.21	11	2.0 (7.8)	0.40	10	1.0 (5.0)	0.68	8		NA
	WBC elevation in 1st week - No	8	**12.1 (13.7)**		7	1.5 (4.4)		6	1.8 (2.1)		6	1.1 (1.1)		0		
	Non-CNS infection - Yes	7	1.5 (3.4)	0.15	10	0.7 (3.3)	0.30	8	1.6 (2.4)	0.54	8	1.0 (3.0)	0.99	5	1.7 (0.2)	0.43
	Non-CNS infection - No	12	6.3 (11.5)		11	1.5 (5.3)		9	2.5 (3.9)		8	1.1 (3.1)		3	4.8 (4.8)	
	Mortaily at discharge - Yes	8	6.7 (13.6)	0.11	5	0.4 (4.2)	0.68	3	**8.9 (5.5)**	**0.006**	3	0.4 (8.2)	0.80	3	0.4 (4.4)	0.32
	Mortaily at discharge - Yes	11	1.5 (6.8)		16	1.0 (3.5)		14	**1.3 (1.6)**		13	1.1 (3.0)		5	1.7 (1.1)	
**TNF-alpha**	Fever in 1st week - Yes	10	0.9 (1.7)	0.46	14	0.9 (0.5)	0.33	12	0.9 (0.0)	0.97	11	0.9 (0.0)	0.92	7	0.9 (0.0)	1.00
**(pg/mL)**	Fever in 1st week - No	9	2.7 (2.2)		7	0.9 (0.0)		5	0.9 (0.0)		5	0.9 (0.0)		1	0.9 (0.0)	
	WBC elevation in 1st week - Yes	11	**0.9 (1.5)**	**0.02**	14	0.9 (0.0)	1.00	11	0.9 (0.0)	1.00	10	0.9 (0.0)	0.75	8		NA
	WBC elevation in 1st week - No	8	**3.0 (4.2)**		7	0.9 (0.0)		6	0.9 (0.0)		6	0.9 (0.0)		0		
	Non-CNS infection - Yes	7	0.9 (1.7)	0.69	10	0.9 (0.0)	0.39	8	0.9 (0.5)	0.91	8	0.9 (0.0)	0.73	5	0.9 (0.0)	0.75

	Non-CNS infection - No	12	1.8 (2.5)		11	0.9 (0.5)		9	0.9 (0.0)		8	0.9 (0.5)		3	0.9 (2.6)	
	Mortaily at discharge - Yes	8	2.6 (2.4)	0.47	5	0.9 (0.0)	0.84	3	0.9 (0.0)	1.00	3	0.9 (0.0)	1.00	3	0.9 (0.0)	1.00
	Mortaily at discharge - Yes	11	0.9 (2.2)		16	0.9 (0.0)		14	0.9 (0.0)		13	0.9 (0.0)		5	0.9 (0.0)	
**IL6**	Fever in 1st week - Yes	10	672 (1706)	0.90	14	**810 (2975)**	**0.04**	12	737 (1280)	0.23	11	353 (55)	0.18	7	606 (2324)	0.50
**(pg/mLx103)**	Fever in 1st week - No	9	1120 (1295)		7	**148 (319)**		5	639 (509)		5	186 (55)		1	74 (0)	
	WBC elevation in 1st week - Yes	11	634 (857)	0.15	14	481 (674)	0.64	11	723 (1565)	0.66	10	195 (1379)	0.56	8		NA
	WBC elevation in 1st week - No	8	1880 (2067)		7	148 (3458)		6	661 (876)		6	319 (240)		0		
	Non-CNS infection - Yes	7	634 (938)	0.34	10	481 (595)	0.60	8	531 (622)	0.09	8	296 (1014)	0.65	5	545 (371)	0.39
	Non-CNS infection - No	12	1092 (1963)		11	397 (3151)		9	351 (1123)		8	246 (2615)		3	2558 (5131)	
	Mortaily at discharge - Yes	8	897 (1723)	0.66	5	372 (385)	0.97	3	11354 (11168)	0.12	3	143 (9306)	0.90	3	74 (5133)	0.57
	Mortaily at discharge - Yes	11	943 (1878)		16	481 (1445)		14	661 (671)		13	295 (550)		5	606 (222)	
**IL8**	Fever in 1st week - Yes	10	850 (7352)	0.90	14	**1352 (2991)**	**0.046**	12	1210 (1350)	1.00	11	1176 (1189)	0.32	7	1040 (3013)	0.50
**(pg/mLx103)**	Fever in 1st week - No	9	955 (1311)		7	**546 (697)**		5	1384 (1428)		5	720 (472)		1	749 (0)	
	WBC elevation in 1st week - Yes	11	667 (566)	0.05	14	960 (1056)	0.69	11	1306 (1467)	1.00	10	1110 (867)	0.43	8		NA
	WBC elevation in 1st week - No	8	2826 (5985)		7	610 (3630)		6	1139 (1824)		6	558 (854)		0		
	Non-CNS infection - Yes	7	667 (788)	0.14	10	985 (1072)	0.76	8	909 (1173)	0.20	8	1110 (777)	1.00	5	907 (149)	0.39
	Non-CNS infection - No	12	1735 (5771)		11	924 (3393)		9	1505 (1251)		8	768 (1943)		3	3904 (3824)	
	Mortaily at discharge - Yes	8	3586 (10001)	0.08	5	867 (844)	0.90	3	1754 (641)	0.24	3	1104 (2184)	0.44	3	749 (3303)	0.39
	Mortaily at discharge - Yes	11	667 (969)		16	960 (2177)		14	1004 (1467)		13	720 (1122)		5	1040 (135)	

**CCL2**	Fever in 1st week - Yes	10	4842 (5506)	0.07	14	7602 (5217)	0.09	12	3727 (1823)	0.33	11	6279 (4657)	0.09	7	6097 (2577)	0.25
**(pg/mLx103)**	Fever in 1st week - No	9	9191 (3666)		7	3379 (2517)		5	5754 (3752)		5	2010 (1497)		1	2538 (0)	
	WBC elevation in 1st week - Yes	11	**4658 (5507)**	**0.005**	14	5882 (4897)	0.53	11	4335 (2260)	0.35	10	1843 (574)	0.04	8		NA
	WBC elevation in 1st week - No	8	**10578 (3305)**		7	3379 (6760)		6	2678 (3998)		6	5106 (3542)		0		
	Non-CNS infection - Yes	7	4658 (4850)	0.14	10	5749 (4160)	1.00	8	4037 (1733)	0.89	8	6353 (3618)	0.07	5	6557 (784)	0.14
	Non-CNS infection - No	12	9419 (5901)		11	3689 (6760)		9	3257 (3418)		8	2087 (1889)		3	3969 (3559)	
	Mortaily at discharge - Yes	8	9091 (7364)	0.54	5	4663 (2701)	0.35	3	3257 (2446)	0.80	3	3934 (3106)	0.70	3	3969 (3478)	0.07
	Mortaily at discharge - Yes	11	6986 (7160)		16	6861 (5640)		14	4037 (3175)		13	2672 (5038)		5	6557 (702)	

P-value: **<0.005** **<0.05** **≥0.05-<0.10**

a Number of patients with cytokine levels at this time point
b IQR - Interquartile Range (75th percentile - 25th Percentile)
c P-values are based on a comparison of distributions using the Mann-Whitney test and are bold if p< 0.05
Abbreviations: WBC, white blood cell; CNS central nervous system.

Table S3. Associations of Age and Glasgow Coma Scale with Cerebrospinal Fluid Cytokine Levels in Intraventricular Hemorrhage Patients by Days Post Hemorrhage Onset.

Days Post Hemorrhage Onset

		1-2 (N=19, 16)[a]		3-4 (N=21, 17)		5-6 (N=17, 14)		7-8 (N=16, 13)		9-10 (N=8, 6)	
**Cytokine**	Radiographic Variables[b]	Corr[b]	P[c]	Corr	P	Corr	P	Corr	P	Corr	P
**IL-1alpha**	Age[d] (Years)	-0.08	0.74	-0.11	0.62	**-0.58**	**0.01**	-0.05	0.85	0.08	0.85
	GCS[d] (1-15)	0.17	0.48	0.04	0.86	0.33	0.20	-0.31	0.24	0.50	0.20
**IL-1beta**	Age (Years)	0.04	0.88	0.31	0.18	-0.20	0.45	0.28	0.29	0.07	0.87
	GCS (1-15)	-0.43	0.07	0.02	0.93	0.04	0.87	-0.22	0.41	0.13	0.75
**IL-10**	Age (Years)	0.24	0.32	0.14	0.56	0.07	0.78	0.19	0.48	0.32	0.44
	GCS (1-15)	-0.39	0.11	-0.42	0.06	-0.37	0.14	-0.28	0.30	0.05	0.91
**TNF-alpha**	Age (Years)	0.12	0.63	-0.01	0.97	**0.57**	**0.02**	0.33	0.21	0.58	0.13
	GCS (1-15)	**0.67**	**0.002**	-0.26	0.26	-0.12	0.65	0.07	0.79	0.00	1.00
**IL6**	Age (Years)	0.08	0.74	0.19	0.41	0.10	0.70	0.25	0.36	0.26	0.53
	GCS (1-15)	-0.39	0.10	0.02	0.95	-0.14	0.60	-0.40	0.12	0.18	0.67
**IL8**	Age (Years)	0.30	0.22	0.28	0.23	0.26	0.32	0.25	0.34	0.22	0.61
	GCS (1-15)	-0.32	0.19	-0.07	0.75	-0.36	0.16	**-0.57**	**0.02**	-0.01	0.98
**CCL2**	Age (Years)	0.10	0.68	0.25	0.28	0.37	0.14	0.22	0.42	0.59	0.13
	GCS (1-15)	-0.25	0.30	0.03	0.89	0.23	0.37	-0.38	0.14	0.24	0.56

P-value **<0.005** **<0.05** **≥0.05-<0.10**

a (N=x,y), where x is number of patients at this time point and y is number of patients for Relative PHE volume, where ICH Volume > 0

b Spearman Correlation.

c P-values are bold if p< 0.05

d Measurements taken at admission

Abbreviations: ICH, intracerebral hemorrhage; IVH, intraventricular hemorrhage; PHE, perihematomal edema; GCS, Glasgow Coma Scale
